# Using real-time visualization system for data-driven decision support to achieve lung protective strategy: a retrospective observational study

**DOI:** 10.1186/s13054-022-04091-0

**Published:** 2022-08-22

**Authors:** How-Yang Tseng, Chieh-Lung Chen, Yu-Chao Lin, Ming-Che Chuang, Wu-Huei Hsu, Wan-Yun Hsiao, Tung-Mei Chen, Min-Tzu Wang, Wei-Chun Huang, Chih-Yu Chen, Biing-Ru Wu, Chih-Yen Tu, Shinn-Jye Liang, Wei-Cheng Chen

**Affiliations:** 1 Division of Pulmonary and Critical Care, Department of Internal Medicine, China Medical University Hospital, No. 2, Yude Road, North District, Taichung, 40402 Taiwan; 2 Department of Internal Medicine, China Medical University Hospital, Taichung, Taiwan; 3 Graduate Institute of Biomedical Sciences and School of Medicine, College of Medicine, China Medical University, Taichung, Taiwan; 4 Critical Medical Center, China Medical University Hospital, Taichung, Taiwan; 5 Department of Respiratory Therapy, China Medical University Hospital, Taichung, Taiwan; 6 Nursing Department, China Medical University Hospital, Taichung, Taiwan; 7 School of Medicine, China Medical University, Taichung, Taiwan; 8 Department of Education, China Medical University Hospital, Taichung, Taiwan

**Keywords:** Real-time visualization, Data-driven decision support, Business intelligence, Acute respiratory distress syndrome, Lung protective strategy, Intensive care unit

## Abstract

**Background:**

Although lung protective strategy and adjunctive intervention are associated with improved survival in patients with acute respiratory distress syndrome (ARDS), the implementation of effective therapies remains low. This study aimed to evaluate whether the use of business intelligence (BI) for real-time data visualization is associated with an improvement in lung protective strategy and adjunctive therapy.

**Methods:**

A retrospective observational cohort study was conducted on patients with ARDS admitted between September 2020 and June 2021 at two intensive care units (ICUs) of a tertiary referral hospital in Taiwan. BI was imported for data visualization and integration to assist in clinical decision in one of the ICUs. The primary outcomes were the implementation of low tidal volume ventilation (defined as tidal volume/predicted body weight ≤ 8 mL/kg) within 24 h from ARDS onset. The secondary outcomes included ICU and hospital mortality rates.

**Results:**

Among the 1201 patients admitted to the ICUs during the study period, 148 (12.3%) fulfilled the ARDS criteria, with 86 patients in the BI-assisted group and 62 patients in the standard-of-care (SOC) group. Disease severity was similar between the two groups. The application of low tidal volume ventilation strategy was significantly improved in the BI-assisted group compared with that in the SOC group (79.1% vs. 61.3%, *p* = 0.018). Despite their ARDS and disease severity, the BI-assisted group tended to achieve low tidal volume ventilation. The ICU and hospital mortality were lower in the BI-assisted group.

**Conclusions:**

The use of real-time visualization system for data-driven decision support was associated with significantly improved compliance to low tidal volume ventilation strategy, which enhanced the outcomes of patients with ARDS in the ICU.

**Supplementary Information:**

The online version contains supplementary material available at 10.1186/s13054-022-04091-0.

## Background

Acute respiratory distress syndrome (ARDS) is a critical condition with high mortality rate. Its average ICU mortality was 35.3%, which increases with disease severity (29.7%, 35.3%, and 42% for mild, moderate, and severe ARDS, respectively) [1]. Patient survival can be improved by adequate ARDS management[2], including early diagnosis, lung protective ventilation strategy (low tidal volume of approximately 6 ml/kg of predicted body weight [PBW] [3] and low plateau pressure < 30 cm H_2_O), administration of neuromuscular blockade [4], and prone positioning in adequate patients with severe disease [5]. Venovenous extracorporeal membrane oxygenation (ECMO) is considered for suitable candidate in experienced ECMO centers [6].

However, the implementation of effective therapies is limited by the lack of recognition of ARDS among clinicians [7, 8]. In the LUNG SAFE study, only half of mild ARDS and three-quarters of severe ARDS were recognized. Low tidal volume ventilation was used in only two-thirds of patients with ARDS, and prone positioning was applied in only 16.3% of patients with severe ARDS [1]. Therefore, exploring a new way to increase clinician recognition and improve ARDS management is crucial.

The health system produces and stores more electronic data than ever before. ICU clinicians are especially exposed to a large number of information from many sources [9], including electronic medical reports, bedside monitors, laboratory results, mechanical ventilator data, and interprofessional recommendations. The use of electronic business intelligence (BI) systems for data analytics can improve the efficiency of the data-driven decision-making process through real-time analytics for data collection, management, and integration [10–12]. Information visualization through BI tools for driving more effective management decisions can also improve the service quality in the medical system [13]. “Microsoft Power BI” is one of the BI tools that gather, process, and turn big data into visually compelling and easy-to-process charts and graphs.

For the above reasons, we designed a real-time visualization dashboard for information integration through Power BI for clinical decision-making assistance based on ARDS protocolized interprofessional cooperation and conducted a retrospective study to evaluate the effect of this technology on ARDS management.

## Methods

### Study design

This retrospective observational study was conducted at two medical ICUs of a tertiary referral hospital in Taiwan. Patients who meet the admission criteria to the ICU were assigned to one of the ICUs based on bed availability. ICU physicians were not involved in the decision of patient assignment. Power BI was applied in one of the ICUs during the study period (BI-assisted group), and standard of care (SOC) was provided in the other group (SOC group). This study was approved by the institutional review board of China Medical University Hospital (CMUH 110-REC1-139). Informed consent was waived because of the retrospective nature of this study and the lack of any personally identifiable information in the gathered data.

### Study participants

Patients who were diagnosed with ARDS and admitted to the ICUs between September 2020 and June 2021 were enrolled. According to the 2012 Berlin definition [14], ARDS was defined as (1) acute onset and rapid progression of lung injury within 1 week, (2) bilateral opacities on chest images that cannot be explained by other lung pathologies, (3) respiratory failure that cannot be fully explained by heart failure and volume overload, and (4) decreased ratio of arterial oxygen tension to inspired fraction of oxygen (PaO_2_/FiO_2_ ratio < 300 mmHg) under a minimum PEEP of 5 cmH_2_O. ARDS severity was categorized as mild, moderate, or severe on the basis of the following criteria: mild (200 mmHg < PaO_2_/FiO_2_ ≤ 300 mmHg), moderate (100 mmHg < PaO_2_/FiO_2_ ≤ 200 mmHg), and severe (PaO_2_/FiO_2_ ≤ 100 mmHg). Final diagnosis was provided by three intensivists after they retrospectively reviewed the clinical data and chest image.

### Business intelligence for real-time visualized data integration

A real-time interactive visualized dashboard was established through Power Business Intelligence—Power BI® (Microsoft Corporation, Redmond, Washington) for ARDS information integration, which enables clinicians to screen the patients with ARDS, monitor the condition of lung protection, and assist in interprofessional discussion and clinical decision in the BI-assisted group.

**Fig. 1:**
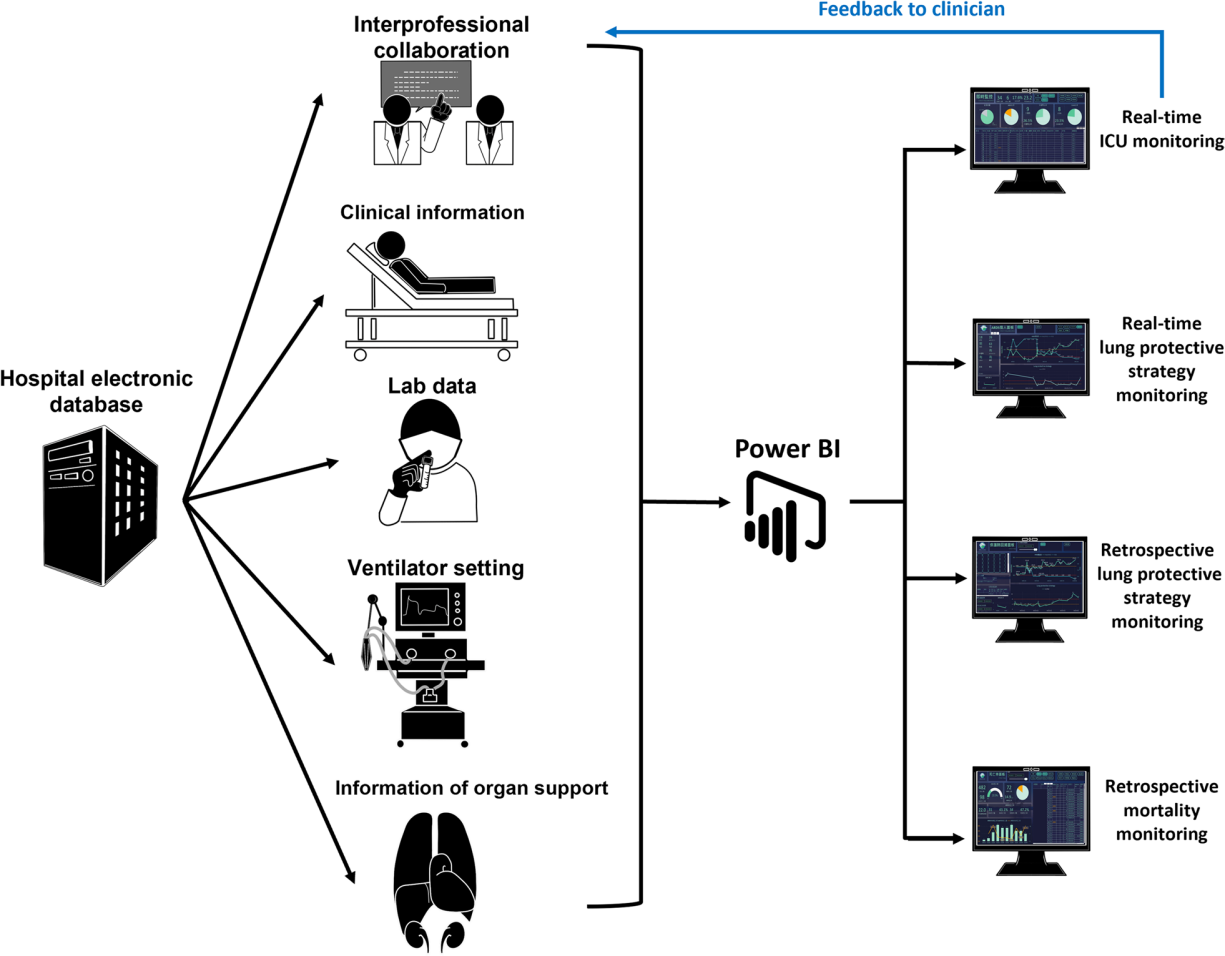
The “Microsoft Power Business Intelligence (BI)” dashboard is connecting directly to the hospital information system for real-time data visualization and integration. The analyses of data will feed back to clinicians for data-driven clinical decision support

The Power BI dashboard is connected directly to the hospital information system (HIS) for real-time and retrospective monitoring (Fig. 1) and automatically detects the International Statistical Classification of Diseases (10th version; ICD-10) coding of ARDS. The real-time dashboard is updated every 15 min, and the retrospective dashboard allows clinicians to access information in any period (Additional file 1: Fig. S1).

The real-time dashboard was used to (1) provide a timely diagnosis of ARDS by rapidly screening the PaO_2_/FiO_2_ ratio in every ICU patient; (2) monitor the in-time percentage of ARDS patients in the ICU; (3) use pie chart to understand the organ support’s current condition, including the use of mechanical ventilation, continuous venovenous hemofiltration, inotropic agents, or ECMO in patients with ARDS; (4) further understand the utility of neuromuscular blockade and prone positioning in patients with moderate-to-severe ARDS; (5) determine the trend of disease severity and lung protective strategy by using the serial data of PaO_2_/FiO_2_ ratio, FiO_2_ and tidal volume/PBW (Vt/PBW) to create a line chart. The retrospective dashboard could display the following information: (1) ARDS incidence, (2) in-ICU and in-hospital mortality rates among patients with ARDS, (3) trend of in-ICU mortality visualized into line and column charts, and (4) quick review of the implementation of lung protective strategy in every patient with ARDS during ICU admission presented as a line chart.

### Education for healthcare providers and protocolized care of ARDS management

Education was imparted to healthcare providers including intensivists, residents, respiratory therapists, and critical care nurses in both groups via (1) the ARDS protocol (Additional file 1: Table S1) and bedside checklists, (2) lectures and online educational materials, (3) case-based simulation training for the junior staff, and (4) interprofessional discussion and collaboration in weekly ward round and bedside tutorials.

### Clinical data collection, clinical assessments, and efficacy evaluations

The following data were collected: demographic and clinical information, including gender, age, etiology and ARDS severity at the time of ARDS diagnosis, and ventilator parameters, including mode of ventilator, tidal volume, and PEEP at 24 h post-ARDS diagnosis. Comorbidities were computed with the modified Charlson comorbidity index. Illness severity upon ICU admission was assessed using Acute Physiology and Chronic Health Evaluation (APACHE) II score, which was calculated using the patient data collected at the time of ICU admission.

The primary outcome was low tidal volume ventilation (defined as tidal volume/PBW ≤ 8 mL/kg) obtained within 24 h from ARDS onset. The secondary outcome was patient survival as represented by ICU and hospital mortality. Mechanical ventilation duration, ICU length of stay (LOS), and hospital LOS were also recorded. All patients were followed until hospital discharge.

### Statistical analyses

Statistical analyses were completed using SPSS version 25 (SPSS Inc., Chicago, IL, USA). Data were expressed as mean with standard deviations or median and interquartile range (IQR) for variables with or without normal distribution, respectively. Continuous data with normal distributions were analyzed using *t* test. Differences between groups were assessed using Mann–Whitney U test for ordinal data and non-normally distributed data. Categorical variables were presented as numbers and percentages and analyzed using chi-square test. A univariate analysis was used to calculate the odds ratio (OR) of ICU mortality. Significant variables in the univariate analysis and clinically important variables were included in the multivariate regression model. The strength of the association was presented as OR and associated 95% confidence interval (CI). All the tests were two-sided, and a *p* value of < 0.05 indicated a statistically significant difference.

## Results

### Patients and baseline characteristics

Among the 1201 patients admitted to the internal medical ICUs during the study period, 148 fulfilled the ARDS criteria, with 86 patients in the BI-assisted group and 62 patients in the SOC group (Additional file 1: Figure S2). Among these patients, the mean (SD) age was 68.1 years (15.1), 56.8% (84) were elderly (≥ 65 years old), 65.5% (97) were male, and 42.6% (63) had an underlying cancer diagnosis. The median (IQR) modified Charlson score was 5 (3–7), and the APACHE II score at ICU admission was 29 (23–35). Shock, which was defined as a need for vasopressors or inotropic agents at the time of ARDS diagnosis, was reported in 75% of patients. No statistically significant differences in baseline characteristics were observed between the two groups (Table 1).

**Table 1:** Characteristics of patients with acute respiratory distress syndrome

	All (N = 148)	Standard-of-care group (N = 62)	BI-assisted group (N = 86)	*p* value
Male	97 (65.5%)	44 (71%)	53 (61.6%)	0.238
Age, years	68.1 ± 15.1	68.6 ± 15.4	67.7 ± 14.9	0.709
Age ≥ 65	84 (56.8%)	36 (58.1%)	48 (55.8%)	0.785
BW, kg	61.2 ± 12.8	62.2 ± 13.5	60.5 ± 12.2	0.410
BMI, kg/m^2^	23.0 ± 4.1	23.4 ± 4.1	22.7 ± 4.1	0.288
Comorbidities				
Cancer	63 (42.6%)	22 (35.5%)	41 (47.7%)	0.139
Modified Charlson score	5 (3–7)	5 (4–8)	5 (4–6)	0.970
Severity of illness				
APACHE II score	29 (23–35)	29 (21.8–35)	28.5 (23–35)	0.855
Shock	111 (75%)	24 (64.9%)	62 (55.9%)	0.336
ARDS etiology				0.150
Viral pneumonia	4 (2.7%)	1 (1.6%)	3 (3.5%)	
Bacterial pneumonia	73 (49.3%)	28 (45.2%)	45 (52.3%)	
Fungal pneumonia	27 (18.2%)	14 (22.6%)	13 (15.1%)	
Aspiration	12 (8.1%)	8 (12.9%)	4 (4.7%)	
Pneumonia of other etiology	15 (10.1%)	3 (4.8%)	12 (14%)	
Extrapulmonary	17 (11.5%)	8 (12.9%)	9 (10.5%)	
ARDS severity at diagnosis				0.908
Mild	26 (17.6%)	10 (16.1%)	16 (18.6%)	
Moderate	74 (50%)	31 (50%)	43 (50%)	
Severe	48 (32.4%)	21 (33.9%)	27 (31.4%)	

*BW* body weight, *BMI* body mass index, *APACHE* Acute Physiology and Chronic Health Evaluation

### ARDS incidence, etiology, and severity

ARDS represented 12.3% of total ICU admissions, with 14.2% (86 per 605 patients) in the BI-assisted group and 10.4% (62 per 596 patients) in the SOC group. The most common etiology of ARDS was bacterial pneumonia (49.3%). Among the patients, 26 (17.6%) were classified as mild ARDS at initial diagnosis, 74 (50%) as moderate, and 48 (32.4%) as severe. No differences in ARDS severity at initial diagnosis were found between the two groups (Table 1).

### Mechanical ventilation and use of adjunctive measures in ARDS

The most utilized ventilator mode was volume control mode in the BI-assisted group and pressure control mode in the SOC group. The fraction of inspiration O_2_ (FiO_2_) and PEEP at 24 h of ARDS diagnosis were similar in both groups (Table 2).

**Table 2:** Ventilator settings and use of adjunctive measures in patients with acute respiratory distress syndrome

	All (N = 148)	Standard-of-care group (N = 62)	BI-assisted group (N = 86)	*p* value
Ventilator settings				
Ventilator mode				**0.002**
PC^a^	78 (52.7%)	44 (71%)	34 (39.5%)	
VC^a^	49 (33.1%)	12 (19.4%)	37 (43%)	
PRVC	10 (6.8%)	2 (3.2%)	8 (9.3%)	
APRV	11 (7.4%)	4 (6.5%)	7 (8.1%)	
Vt/PBW (mL/kg)	7.1 (6.3–8.3)	7.5 (6.5–9.5)	6.9 (6.1–7.7)	**0.014**
FiO2	0.5 (0.4–0.7)	0.5 (0.4–0.7)	0.5 (0.4–0.7)	0.392
PEEP	10 (8–12)	10 (8–12)	10 (8–12)	0.368
Adjunctive measures				
Prone	62 (41.9%)	24 (38.7%)	38 (44.2%)	0.505
NMB	103 (69.6%)	43 (69.4%)	60 (69.8%)	0.957
ECMO	6 (4.1%)	3 (4.8%)	3 (3.5%)	0.681

^a^The adjusted standardized residual was greater than 2 which indicates the column proportions were significantly different at *p* < 0.05 level

*PC* pressure control, *VC* volume control, *PRVC* pressure-regulated volume control, *APRV* airway pressure release ventilation, *Vt* tidal volume, *FiO*2 fraction of inspiration O2, *PBW* predicted body weight, *PEEP* positive end-expiratory pressure, *NMB* neuromuscular blockade, *ECMO* extracorporeal membrane oxygenation

**Fig. 2:**
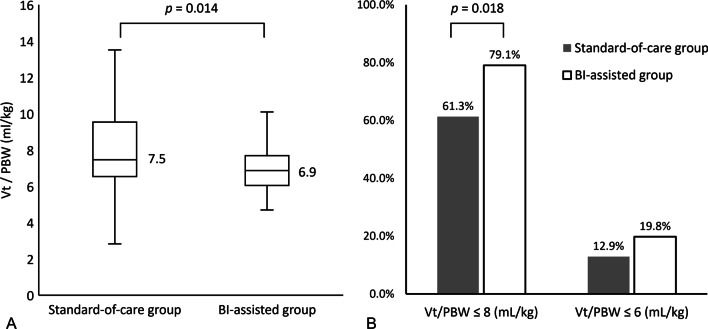
Compliance of low tidal volume ventilation between BI-assisted group and standard-of-care (SOC) group. **A** The median tidal volume/predicted body weight (PBW) was 6.9 mL/kg (IQR 6.1–7.7 mL/kg) in the BI-assisted group, significantly lower than 7.5 mL/kg (IQR 6.5–9.5 mL/kg) in the SOC group, *p* = 0.014 **B** Application of ventilator setting of tidal volume per PBW ≤ 8 mL/kg at 24 h of ARDS diagnosis was significantly better in the BI-assisted group than the SOC group (79.1% vs. 61.3%, *p* = 0.018). More patients received tidal volume per PBW less than 6 mL/kg in the BI-assisted group (19.8% vs. 12.9%, *p* = 0.271)

The median tidal volume was 6.9 mL/kg (IQR 6.1–7.7 mL/kg) of PBW in the BI-assisted group, which was significantly lower than the 7.5 mL/kg (IQR 6.5–9.5 mL/kg) in the SOC group (*p* = 0.014) (Fig. 2A). As represented by the ventilator setting of tidal volume/PBW ≤ 8 mL/kg, the implementation of low tidal ventilation was significant better in the BI-assisted group than in the SOC group (79.1% vs. 61.3%, *p* = 0.018). Compared with those in the SOC group, more patients received tidal volume per PBW ≤ 6 in the BI-assisted group (19.8% vs. 12.9%, *p* = 0.271) (Fig. 2B).

The use of neuromuscular blockade was similar between the two groups (69.8% vs. 69.4%). More patients received prone positioning as adjunctive measure in the BI-assisted group than in the SOC group (44.2% vs. 38.7%), but the difference was not statistically significant (Table 2).

### Comparison of low tidal ventilation and disease severity

**Fig. 3:**
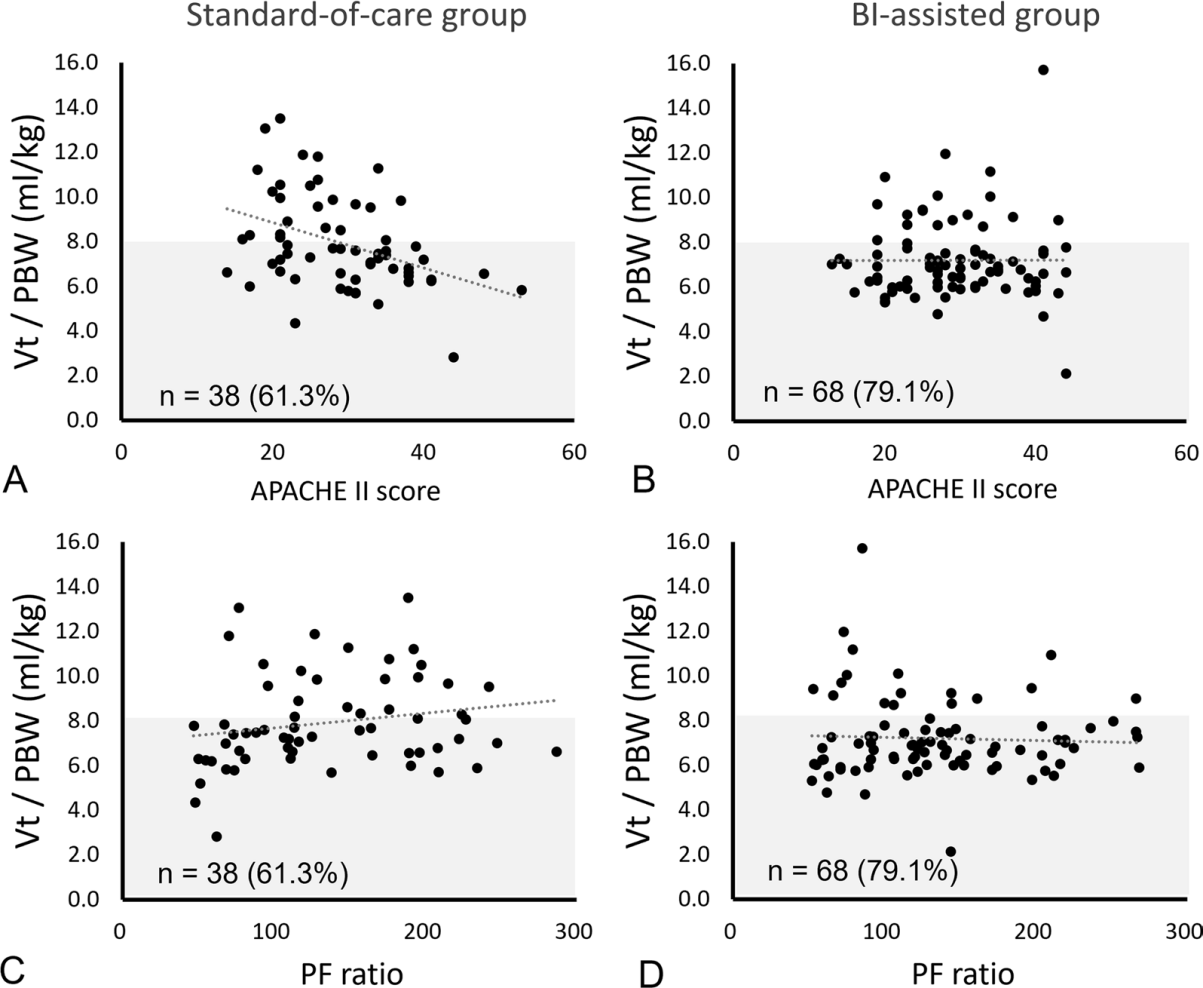
Relationship of tidal volume settings between different disease severity entities. The disease severity was represented by Acute Physiology and Chronic Health Evaluation II (APACHE II) score and PaO2/FiO2 ratio. **A**, **C** In the standard-of-care group, patients with lower APACHE II score and higher PaO2/FiO2 ratios were more likely to receive higher tidal volume setting. (**B**, **D**) In the BI-assisted group, patients received low tidal volume ventilation irrespective of APACHE II score and PaO2/FiO2 ratio

The distribution of tidal volume/PBW vs. APACHE II score and PaO_2_/FiO_2_ ratio is presented in Fig. 3. In the SOC group, the patients with low APACHE II score and high PaO_2_/FiO_2_ ratios were less likely to receive low tidal volume ventilation (Fig. 3A, C). Irrespective of disease severity and ARDS severity, compliance to low tidal volume ventilation strategy was better in the BI-assisted group (Fig. 3B, D).

### ARDS outcomes and prognostic factors

The overall ICU and hospital mortality rates were 47.3% and 56.8%, respectively. The ICU mortality rate was 39.5% in the BI-assisted group, which was significantly lower than that in the SOC group (58.1%). The hospital mortality rate was also significantly lower in the BI-assisted group than in the SOC group (48.8% vs. 67.7%, *p* = 0.022). The patients in the SOC group received a longer duration of mechanical ventilation and had a longer ICU LOS than those in the BI-assisted group, but the difference was not statistically significant. Meanwhile, the patients in the BI-assisted group had a longer hospital LOS compared with those in the SOC group (Table 3).

**Table 3:** Comparison of clinical outcomes between standard-of-care group and BI-assisted group

	All (N = 148)	Standard-of-care group (N = 62)	BI-assisted group (N = 86)	*p* value
Duration of IMV, day	11 (5–26)	13 (6–26.8)	10 (5–26)	0.741
ICU mortality	70 (47.3%)	36 (58.1%)	34 (39.5%)	0.026
Hospital mortality	84 (56.8%)	42 (67.7%)	42 (48.8%)	0.022
ICU LOS, day	12 (6.3–19.8)	13 (8–23.3)	10 (5–15.3)	0.055
Hospital LOS, day	26 (14–49.8)	23 (12.8–39.3)	28 (14.8–50.5)	0.221

*IMV* invasive mechanical ventilation, *ICU* intensive care unit, *LOS* length of stay

After adjusting for possible confounders and using logistic regression analysis, BI assistance was an independent good prognostic factor for ICU survival (OR 0.45; 95% CI 0.22–0.92), whereas a higher APACHE II score was independently associated with increased ICU mortality (OR 1.05; 95% CI 1.00–1.10). The prognostic effects of BI assistance and APACHE II score remained consistent in the regression analysis for hospital outcomes (Additional file 1: Table S2).

## Discussion

The study is the first to focus on the clinical effect of BI on improving lung protective strategy in patients with ARDS. Our results revealed that using BI for real-time data visualization and integration to assist clinical decision-making on lung protective strategy led to significantly better compliance to low tidal volume ventilation, thus further improving the outcomes of patients with ARDS in the ICU. This work indicates the potential for enhancing the management of patients with ARDS.

Our study showed that ARDS accounted for 12.3% of ICU patients with seasonal variation of 6.2%–15.5%. Bellani et al. conducted a large international, multicenter, prospective cohort study and found that ARDS constituted about 10% of ICU admissions (1). With the real-time data-visualized dashboard, clinicians could keep tract of the updated percentage of patients with ARDS in the ICU anytime. Frohlich et al. reported that ARDS in the ICU was underrecognized by clinicians [7]. Needham et al. showed that early ARDS recognition is crucial for improving patient survival [8]. However, the clinical recognition of ARDS remains low. Bellani et al. reported that only 60% of ARDS cases were diagnosed. Although the clinical recognition rate increases with ARDS severity, severe ARDS was recognized in less than 80% of cases [1]. Easily and carefully monitoring the potential ARDS cases could lead to timely diagnosis, an important first step in improving the outcome of patients with ARDS in the ICU.

Our data revealed that an exact low tidal volume is more easily achieved with the support of real-time data visualization dashboard. Among the patients in the BI-assisted group, 79.1% received a tidal volume less than 8 ml/kg PBW and 19.8% achieved a tidal volume less than 6 ml/kg PBW. However, among the patients in the SOC group, only 61.3% received a tidal volume less than 8 ml/kg PBW, which was similar to the LUNG SAFE study findings [1]. Brower et al. reported that mechanical ventilation with low tidal volume in patients with ARDS can reduce mortality and increase ventilator-free days [3]. Needham et al. revealed that high tidal volume ventilation shortly after ARDS leads to high ICU mortality and emphasized the timing of low tidal volume ventilation [8]. With BI intervention, the median tidal volume/PBW was significantly decreased compared with that in the SOC group (6.9 vs. 7.5 mL/kg,* p* = 0.014). Regardless of disease severity, patients in the BI-assisted group tended to reach low tidal volume as indicated by their PaO_2_/FiO_2_ ratio and APACHE II score. In the SOC group, ARDS may be underrecognized, or the low tidal volume ventilation strategy might be less followed, particularly in patients with less disease severity.

In our study, “low tidal volume” was used as a target and an easy-to-understand concept of lung protection for intensivists, critical care pulmonologists, and every professional involved in ICU daily care. Amato et al. disclosed that reduced driving pressure is an index that is strongly associated with survival [15]. Meanwhile, the LUNG SAFE study revealed no superiority of driving pressure over plateau pressure in improving survival [1, 16]. Despite the ongoing discussions about the key index of ventilator’s setting, the concept of lung protective strategies, such as low tidal volume (approximately 6 mL/kg predicted body weight) and limited plateau pressure (< 30 cmH_2_O), has become a standard of care in ARDS management [2]. Nevertheless, the LUNG SAFE study reported that approximately 35% of patients with ARDS received a tidal volume of more than 8 mL/kg PBW [1]. With the assistance of real-time visualization system, clinicians could stay alert and easily monitor the dynamic severity trend of ARDS and compliance to low tidal volume ventilation strategy, which is important during the high prevalence season of ARDS or pandemics, such as the coronavirus disease 2019, when clinicians have to manage many patients with ARDS at the same time.

Our study found that the prone position was used in 41.9% of patients with ARDS, which was higher than the LUNG SAFE study, that the prone position was used in 7.9% of patients with ARDS [1]. In patients with severe ARDS, the use of adjunctive measures including neuromuscular blocking agents and prone positioning improved outcomes [2, 4, 5]. However, the technical aspects of safely and promptly implementing prone positioning in the ICU are not simple and, to some degree, limit universality. Therefore, we assembled a coordinated team that could perform prone positioning at any time through conducting a series of education for healthcare providers and protocolized care of ARDS management to improve this problem. Our results revealed no differences in the use of neuromuscular blockers and prone positioning for ARDS between the two groups. This phenomenon occurred because the clinician recognition rates increased with disease severity, and severe cases were frequently diagnosed. However, despite timely adjunctive measure interventions, the low tidal volume ventilation should be the first approach in ARDS management. Adjunctive intervention, such as the use of neuromuscular blockade, should be managed with low tidal volume ventilation [2, 4].

The disease severity among the patients in our study is high, of which 56.8% of patients were older than 65 years old and 42.6% had a cancer history. The mean APACHE II score was 29, and shock occurred in 75% of patients at the time of ARDS diagnosis. ARDS is related to high mortality, and variations were noted among different centers. Kao et al. showed that patients aged ≥ 65 years old with ARDS had poor outcomes than the patients < 65 years old [17]. The ALIEN study revealed an ICU mortality of 42.7% and hospital mortality of 47.8% with a mean APACHE II score of 21.6 [18]. In our study, the ICU and the hospital mortality rate was 39.5% and 48.8% in the BI-assisted group, respectively, which was significantly lower than that in the SOC group. Regardless of the severe and complicated disease status, strictly following the current standard of care of ARDS management could lead to improvements in ICU and hospital mortality rates.

The benefits of BI for real-time visualized and integrated information that affects the management of ARDS are data-driven decision-making and transparency, which can be used as a strong way to provide accountability for performance improvement [13]. The barriers to low tidal volume ventilation include physician's concerns of contraindication or complications of low tidal volume ventilation [19, 20]. Poor communication and coordination between the multi-disciplinary teams are the reasons for poor application of physician's order [21]. Through the education for healthcare providers and protocolized care, the awareness and knowledge of ARDS management among the ICU team could be enhanced, which further promotes behavioral changes [22]. With the feedback from multi-disciplinary ICU teams, the team-based interventions could be complementary to each other. Additionally, Sjoding reported that behavioral economic strategy would also influence the clinicians’ decision-making because their behavior is often affected by cognitive, social, and emotional factors [23]. The introduction of real-time visualization dashboard through BI could provide a visual suggestion to every ICU professional to achieve the lung protective strategy.

This study has some limitations. First, given the retrospective design, some variables were not recorded, such as driving pressure. The driving pressure cannot be measured in all modes, for example, the airway pressure release ventilation (APRV) and pressure-regulated volume control (PRVC) modes. Besides, the targets of lung protection were not similar in different modes. Because no evidence shows that the ventilator mode is associated with better outcomes [24], the mode of ventilation was determined by the clinical team. In our study, the pressure control mode was used in 52.7%, volume control mode in 33.1%, pressure-regulated volume control mode in 6.8%, and airway pressure release ventilation mode in 7.4% of the patients. Therefore, the driving pressure and plateau pressure were not routinely measured. And we used “tidal volume” as a target, which could overcome the limitation of the mode difference. Second, the sample size was relatively small; thus, a randomized controlled trial with larger sample size is necessary to provide higher-quality data and minimize potential bias. Finally, the improvement in ARDS management is due not only to the use of real-time visualization system for data-driven decision support but also to the advancement of the consensus of ARDS recognition and lung protective strategy among ICU members through education and protocolized care in ARDS. Nevertheless, this method offers an easy and convenient way to improve the quality of ARDS care.

## Conclusion

Using the real-time visualization system for data-driven decision support that allows clinicians to strictly follow the lung protective strategy and adjunctive therapies results in improved compliance to low tidal volume ventilation strategy and ICU outcomes in patients with ARDS.

## Supplementary Information

**Additional file 1**: **Fig. S1**. The real-time and retrospective interactive visualized dashboard was established through “Microsoft Power BI” for ARDS patients monitoring and data-driven decision support. **Fig. S2**. Flowchart of enrolled subjects. **Table S1.** The ARDS “Lung protective strategy” protocol in the medical intensive care units of China Medical University Hospital. **Table S2**. Logistic regression analysis of ICU and hospital mortality determinants.

## Data Availability

The datasets used and analyzed during the current study are available from the corresponding author on reasonable request.

## Abbreviations

ARDS: Acute respiratory distress syndrome
APACHE II: Acute Physiology and Chronic Health Evaluation II
APRV: Airway pressure release ventilation
BI: Business intelligence
CVVH: Continuous venovenous hemofiltration
COVID-19: Coronavirus disease 2019
ICU: Intensive care unit
LOS: Length of stay
IQR: Interquartile range
OR: Odds ratio
PBW: Predicted body weight
PEEP: Positive end-expiratory pressure
SOC: Standard of care
ECMO: Extracorporeal membrane oxygenation
