# Supplementary material for: Using real-time visualization system for data-driven decision support to achieve lung protective strategy: a retrospective observational study
Source: Crit Care. 2022 Aug 22;26:253. doi: 10.1186/s13054-022-04091-0 (PMC9395891; doi:10.1186/s13054-022-04091-0)
Supplement: Supplementary file 1 — Additional file 1: Fig. S1. The real-time and retrospective interactive visualized dashboard was established through “Microsoft Power BI” for ARDS patients monitoring and data-driven decision support. Fig. S2. Flowchart of enrolled subjects. Table S1. The ARDS “Lung protective strategy” protocol in the medical intensive care units of China Medical University Hospital. Table S2. Logistic regression analysis of ICU and hospital mortality determinants. [file 13054_2022_4091_MOESM1_ESM.docx]

**Using** **real-time visualization system for data-driven decision support to achieve lung protective strategy**

**Additional File 1**

**Table of Contents**

**Figure S1:**

The Real-time and retrospective interactive visualized dashboard was established through “Microsoft Power BI” for ARDS patients monitoring and data-driven decision support.

**Figure S2:** Flowchart of enrolled subjects

**Table S1:** The ARDS “Lung protective strategy” protocol in the medical intensive care units of China Medical University Hospital.

**Table S2:** Logistic regression analysis of ICU and hospital mortality determinants.

**Figure S1:**

The Real-time and retrospective interactive visualized dashboard was established through “Microsoft Power BI” for ARDS patients monitoring and data-driven decision support.


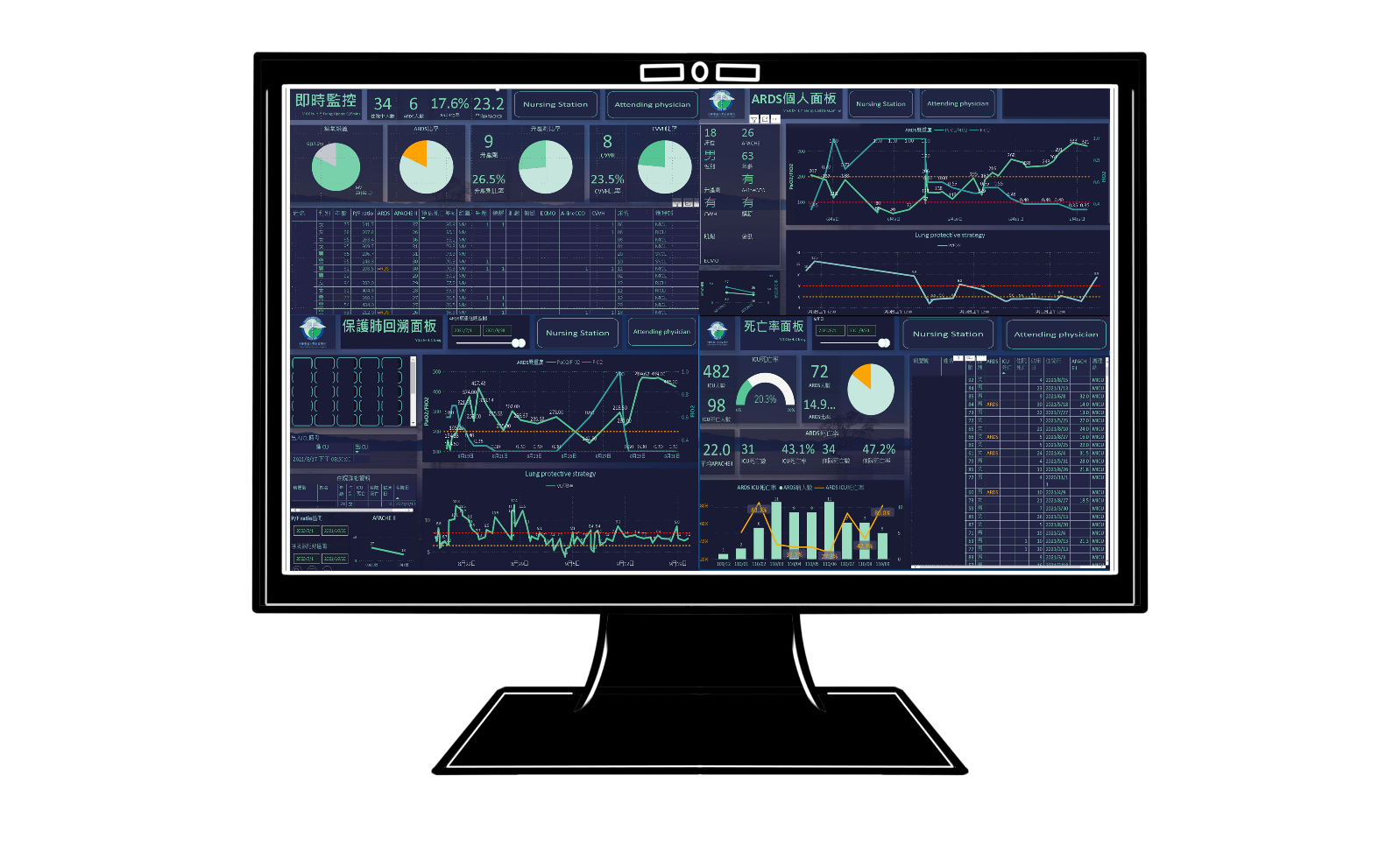


**Figure S2:** Flowchart of enrolled subjects

**
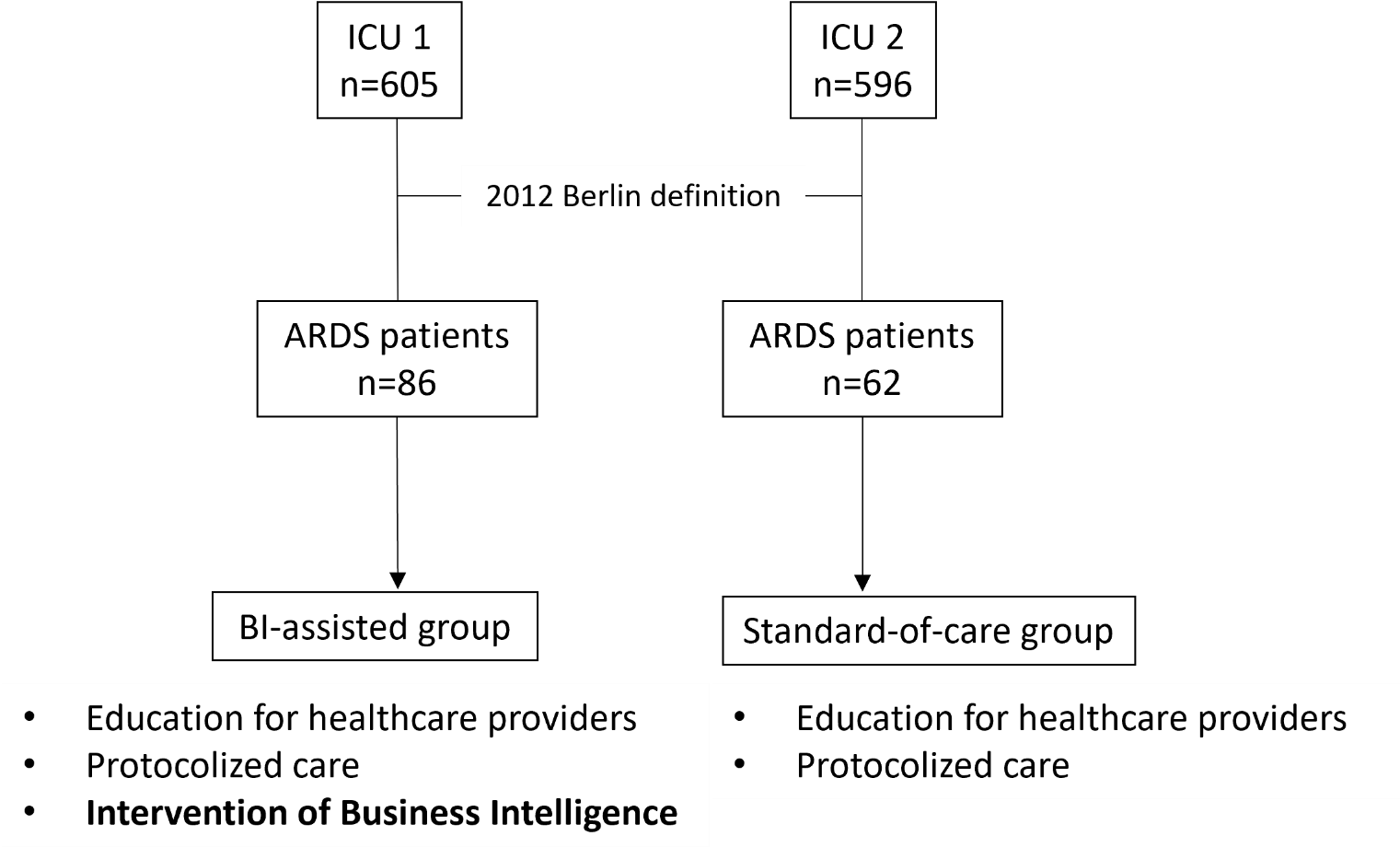
**

**Table S1:** The ARDS “Lung protective strategy” protocol in the medical intensive care units of China Medical University Hospital.

**CMUH ARDS「Lung Protective Strategy」Protocol^1^**

| ARDS 2012 Berlin definition^2^ |
| --- |
| - Onset：Within 1 week of insult, new (within 7 days) or worsening respiratory symptoms - Images：Bilateral airspace disease on chest x-ray or computed tomography not fully explained by effusions, lobar or lung collapse, or nodules - Cannot fully explained by heart failure and volume overload - Hypoxemia- PaO_2_/FIO_2_ ≤ 300 mmHg under PEEP ≥ 5 cmH_2_O   Mild: PaO_2_/FIO_2_ ≤ 300 mmHg  Moderate: PaO_2_/FIO_2_ ≤ 200 mmHg  Severe: PaO_2_/FIO_2_ ≤ 100 mmHg |

**PaO_2_/FIO_2_ ≤ 300 mmHg**

| Lung protective strategy ^3^ |
| --- |
| - Tidal volume 6 mL/kg PBW* (4-8 mL/kg) within 4 hours   ***PBW (**predicted body weight)  Male= 50+0.91(Height-152.4)  Female=45.5+0.91(Height-152.4)   - Pplat ≤ 30 cmH2O - Adequate PEEP (as table) - Goal (clinical judge)   - Oxygenation: PaO_2_ 55-80 mm Hg or SpO_2_ 88%-95%   - Ventilation: pH ≥ 7.25, Respiratory rate ≤ 35 breaths per minute - Sedation if needed for ventilator synchronization - F/u arterial gas q6h & 0.5-1 hr after adjustment or dependence of ventilator |

| Use these FIO_2_/PEEP combinations to achieve oxygenation goal | | | | | | | | |
| --- | --- | --- | --- | --- | --- | --- | --- | --- |
| FiO_2_ | 0.3 | 0.4 | 0.5 | 0.6 | 0.7 | 0.8 | 0.9 | 1.0 |
| PEEP | 5 | 5-8 | 8-10 | 10 | 10-14 | 14 | 14-18 | 18-24 |

**PaO_2_/FIO_2_ < 150 mmHg**

| Neuromuscular blocker^4^ | |
| --- | --- |
| - Timing: Early-first 48h of moderate-severe ARDS (PaO_2_/FIO_2_ < 150 mmHg under PEEP of 5 cmH_2_O) - Continuous IV infusion for ventilator synchronization (RASS has to be -4~-5 !) | |
| Prone Position^5^ | |
| - Timing: PaO_2_/FIO_2_ < 150 mmHg under PEEP of 5 cmH_2_O and FIO_2_ ≥ 0.6 - Duration: At least 16 hours a day - Stop criteria: - Improvement of oxygenation   In the supine position, under PEEP ≤ 10 cmH_2_O and FIO_2_ ≤ 0.6, PaO_2_/FIO_2_ ≥ 150 mmHg at least 4 hours   - Worsening of oxygenation   Decrease in P/F ratio ≥ 20% or SpO_2_<85% under FIO_2_ 100% for 5mins   - Complications: massive facial or oral bleeding, dislocation of line or tube… | Supine position **P/F > 150 for 4 hr**  (FIO_2_≤0.6, PEEP≤10)?  Keep supine position  Supine position P/F < 150?  **Prone position** at least **16 hr** a day  Yes  Yes  No  Yes |

**PaO_2_/FIO_2_ < 80 mmHg**

| ECMO^6^ |
| --- |
| - Timing: Early if no contraindications - Hypoxemia FIO_2_>0.8 & optimal care as above   P/F ratio <80 for 6 hr  P/F ratio <50 for 3 hr   - Hypercapnia , despite RR ≤ 35 and Pplat ≤ 30 cmH2O   PaCO_2_>60mmHg & pH<7.25 for 6H   - During ECMO: Ultra-protective ventilation: Tidal volume 4 mL/kg PBW   (PEEP: 10 cmH_2_O / Driving pressure: 10 cmH_2_O / RR:10 bpm / FIO_2_ 0.4)  **PBW and Tidal Volume for Females** |

| Height (cm) | PBW | 4ml | 5ml | 6ml | 7ml | 8ml |
| --- | --- | --- | --- | --- | --- | --- |
| 130 | 25.2 | 101 | 126 | 151 | 177 | 202 |
| 135 | 29.7 | 119 | 149 | 178 | 208 | 238 |
| 140 | 34.2 | 137 | 171 | 206 | 240 | 274 |
| 142 | 36.1 | 144 | 180 | 216 | 253 | 289 |
| 144 | 37.9 | 151 | 189 | 227 | 265 | 303 |
| 146 | 39.7 | 159 | 199 | 238 | 278 | 318 |
| 148 | 41.5 | 166 | 208 | 249 | 291 | 332 |
| 150 | 43.3 | 173 | 217 | 260 | 303 | 347 |
| 152 | 45.1 | 181 | 226 | 271 | 316 | 361 |
| 154 | 46.9 | 188 | 235 | 282 | 329 | 376 |
| 156 | 48.8 | 195 | 244 | 293 | 341 | 390 |
| 158 | 50.6 | 202 | 253 | 303 | 354 | 405 |
| 160 | 52.4 | 210 | 262 | 314 | 367 | 419 |
| 162 | 54.2 | 217 | 271 | 325 | 379 | 434 |
| 164 | 56.0 | 224 | 280 | 336 | 392 | 448 |
| 166 | 57.8 | 231 | 289 | 347 | 405 | 463 |
| 168 | 59.6 | 239 | 298 | 358 | 417 | 477 |
| 170 | 61.4 | 246 | 307 | 369 | 430 | 491 |
| 172 | 63.2 | 253 | 316 | 379 | 443 | 506 |
| 174 | 65.1 | 260 | 325 | 390 | 455 | 520 |
| 176 | 66.9 | 268 | 334 | 401 | 468 | 535 |
| 178 | 68.7 | 275 | 343 | 412 | 481 | 549 |
| 180 | 70.5 | 282 | 352 | 423 | 493 | 564 |

| Height (cm) | PBW | 4ml | 5ml | 6ml | 7ml | 8ml |
| --- | --- | --- | --- | --- | --- | --- |
| 140 | 38.8 | 155 | 194 | 233 | 271 | 310 |
| 145 | 43.3 | 173 | 216 | 260 | 303 | 346 |
| 150 | 47.8 | 191 | 239 | 287 | 335 | 383 |
| 152 | 49.6 | 199 | 248 | 298 | 347 | 397 |
| 154 | 51.4 | 206 | 257 | 309 | 360 | 412 |
| 156 | 53.3 | 213 | 266 | 320 | 373 | 426 |
| 158 | 55.1 | 220 | 275 | 330 | 385 | 441 |
| 160 | 56.9 | 228 | 284 | 341 | 398 | 455 |
| 162 | 58.7 | 235 | 293 | 352 | 411 | 470 |
| 164 | 60.5 | 242 | 303 | 363 | 424 | 484 |
| 166 | 62.3 | 249 | 312 | 374 | 436 | 499 |
| 168 | 64.1 | 257 | 321 | 385 | 449 | 513 |
| 170 | 65.9 | 264 | 330 | 396 | 462 | 527 |
| 172 | 67.7 | 271 | 339 | 406 | 474 | 542 |
| 174 | 69.6 | 278 | 348 | 417 | 487 | 556 |
| 176 | 71.4 | 285 | 357 | 428 | 500 | 571 |
| 178 | 73.2 | 293 | 366 | 439 | 512 | 585 |
| 180 | 75 | 300 | 375 | 450 | 523 | 600 |
| 182 | 76.8 | 307 | 384 | 461 | 538 | 614 |
| 184 | 78.6 | 314 | 393 | 472 | 550 | 629 |
| 186 | 80.4 | 322 | 402 | 483 | 563 | 643 |
| 188 | 82.2 | 329 | 411 | 493 | 576 | 658 |
| 190 | 84 | 336 | 420 | 504 | 588 | 672 |

**PBW and Tidal Volume for Males**

**Reference**

1. Fan et al, Acute Respiratory Distress Syndrome-Advances in Diagnosis and Treatment. JAMA. 2018;319(7):698-710
2. ARDS definition task force. Acute Respiratory Distress Syndrome-The Berlin Definition. JAMA. 2012;307(23):2526-2533
3. The ARDS network, Ventilation with Lower Tidal Volumes as Compared with Traditional Tidal Volumes for Acute Lung Injury and Acute Respiratory Distress Syndrome. N Engl J Med 2000;342:1301-8.
4. Papazian et al, Neuromuscular Blockers in Early Acute Respiratory Distress Syndrome. N Engl J Med 2010;363:1107-16.
5. Guerin et al, Prone Positioning in Severe Acute Respiratory Distress Syndrome. N Engl J Med 2013;368:2159-68.
6. Combes et al, Extracorporeal Membrane Oxygenation for Severe Acute Respiratory Distress Syndrome. N Engl J Med 2018;378:1965-75

**Table S2:** Logistic regression analysis of ICU and hospital mortality determinants.

|  | | ICU mortality | | | |  | Hospital mortality | | | |
| --- | --- | --- | --- | --- | --- | --- | --- | --- | --- | --- |
|  |  | Univariate analysis | | Multivariate analysis ^a^ | |  | Univariate analysis | | Multivariate analysis ^b^ | |
| **Variables** | | **OR (95% CI)** | ***p* value** | **OR (95% CI)** | ***p* value** |  | **OR (95% CI)** | ***p* value** | **OR (95% CI)** | ***p* value** |
| Age ≥ 65 | | 1.15 (0.60-2.21) | 0.673 | 1.04 (0.51-2.13) | 0.921 |  | 1.04 (0.54-2.00) | 0.914 | 0.88 (0.41-1.90) | 0.741 |
| Cancer | | 2.00 (1.03-3.88) | **0.04** | 2.04 (0.99-4.19) | 0.053 |  | 2.05 (1.04-4.02) | **0.037** | 1.99 (0.94-4.24) | 0.073 |
| APACHE II score | | 1.05 (1.01-1.10) | **0.014** | 1.05 (1.00-1.10) | **0.045** |  | 1.06 (1.02-1.11) | **0.008** | 1.07 (1.01-1.12) | **0.012** |
| Causes of ARDS | |  | |  | |  |  |  |  |  |
|  | Extrapulmonary | *Reference* | |  | |  | *Reference* | | *Reference* | |
|  | Intrapulmonary | 2.36 (0.79-7.09) | 0.125 |  |  |  | 2.70 (0.94-7.74) | 0.065 | 2.97 (0.86-10.26) | 0.085 |
| Shock | | 1.95 (0.90-4.21) | 0.090 | 1.36 (0.57-3.25) | 0.489 |  | 1.79 (0.85-3.79) | 0.128 |  |  |
| ARDS severity at diagnosis | |  | |  | |  |  |  |  |  |
|  | Mild | *Reference* | |  | |  | *Reference* | | *Reference* | |
|  | Moderate | 1.52 (0.62-3.75) | 0.363 |  |  |  | 2.15 (0.87-5.33) | 0.097 | 1.99 (0.71-5.57) | 0.188 |
|  | Severe | 0.97 (0.37-2.56) | 0.957 |  |  |  | 1.17 (0.45-3.04) | 0.752 | 0.76 (0.26-2.25) | 0.618 |
| Vt/PBW ≤ 8 | | 0.66 (0.32-1.35) | 0.254 | 0.59 (0.27-1.30) | 0.192 |  | 0.65 (0.31-1.35) | 0.246 | 0.72 (0.31-1.68) | 0.451 |
| BI assistance | | 0.47 (0.24-0.92) | **0.027** | 0.45 (0.22-0.92) | **0.029** |  | 0.46 (0.23-0.90) | **0.023** | 0.37 (0.17-0.80) | **0.012** |
| a: Hosmer and Lemeshow test: *p*=0.14, b: Hosmer and Lemeshow test: *p*=0.508 | | | | | | | | | | |
